# To the Editors of the Medical and Physical Journal

**Published:** 1801-07

**Authors:** 

**Affiliations:** Oxford Street


### *To the Editors of the Medical and Phyfical Journal.*

GENTLEMEN,

IT was with pleafure I read in your ufeful Journal an account of the origin of the Medical School at Edinburgh, and I have no doubt that it would give great fatisfaction to many of your readers, efpecially the junior part, who may wifh to compleat their medical education at fome univerfity, to have the plan of proceeding at Edinburgh, Glafgow, St. Andrews, and Aberdeen. A principal point is the time neceffary to attend at each place, before a diploma can be obtained, and alfo the forms neceffary to be complied with previous to its attainment; a ftatement of the ufual fees, and whether teftimonials of attendance to the ftudy of phyfic in London would be admitted. If this is not incompatible with the intention of your excellent work, I am convinced it would be very acceptable information to the junior part of your readers, in affifting them to arrange the plan of their ftudies. This idea has prompted me to lay my fentiments before you; and if you view it in the fame light as I do, from the perfonal knowledge I have of your candour and readinefs to communicate any medical information, I flatter myfelf I fhall foon fee a fatisfactory account of thefe particulars; but fhould the profeffional engagements of the Editors prevent their drawing up fuch an account, perhaps on this hint fome other perfon would ftep forward.

I am, &c

*Oxford Street, June* 20, 1801. SILURIENSIS.

